# “Loved ones are not ‘visitors' in a patient's life”—The importance of including loved ones in the patient's hospital stay: An international Twitter study of #HospitalsTalkToLovedOnes in times of COVID-19

**DOI:** 10.3389/fpubh.2023.1100280

**Published:** 2023-01-26

**Authors:** Mojca Hriberšek, Fabian Eibensteiner, Lorenz Kapral, Anna Teufel, Faisal A. Nawaz, Merisa Cenanovic, Chandragiri Siva Sai, Hari Prasad Devkota, Ronita De, Rajeev K. Singla, Emil D. Parvanov, Christos Tsagkaris, Atanas G. Atanasov, Eva Schaden

**Affiliations:** ^1^Ludwig Boltzmann Institute Digital Health and Patient Safety, Medical University of Vienna, Vienna, Austria; ^2^Division of Pediatric Nephrology and Gastroenterology, Department of Pediatrics and Adolescent Medicine, Comprehensive Center for Pediatrics, Medical University of Vienna, Vienna, Austria; ^3^Department of Psychiatry, Al Amal Psychiatric Hospital, Dubai, United Arab Emirates; ^4^Independent Researcher, Sarajevo, Bosnia and Herzegovina; ^5^Amity Institute of Pharmacy, Amity University, Noida, India; ^6^Graduate School of Pharmaceutical Sciences, Kumamoto University, Kumamoto, Japan; ^7^Pharmacy Program, Gandaki University, Pokhara, Nepal; ^8^Department of ICMR-NICED Virus Lab, Indian Council of Medical Research-National Institute of Cholera and Enteric Diseases, Kolkata, West Bengal, India; ^9^Institutes for Systems Genetics, Frontiers Science Center for Disease-Related Molecular Network, West China Hospital, Sichuan University, Chengdu, Sichuan, China; ^10^School of Pharmaceutical Sciences, Lovely Professional University, Phagwara, Punjab, India; ^11^Department of Translational Stem Cell Biology, Research Institute of the Medical University of Varna, Varna, Bulgaria; ^12^Faculty of Medicine, University of Crete, Heraklion, Greece; ^13^Department of Biotechnology and Nutrigenomics, Institute of Genetics and Animal Biotechnology of the Polish Academy of Sciences, Magdalenka, Poland; ^14^Department of Anaesthesia, Intensive Care Medicine and Pain Medicine, Medical University of Vienna, Vienna, Austria

**Keywords:** family-centered care, doctor–patient relationship, hospital care, social media, visitation restrictions, patient experience, patient safety, crowdsourcing

## Abstract

**Background:**

Hospitals are institutions whose primary task is to treat patients. Family-centered care, which considers loved ones as equal partners in patient care, has been gaining recognition in the adult care setting. Our aim was to record experiences of and opinions on communication between hospital-based healthcare providers and patients' loved ones, related but not limited to the rigorous mitigation measures implemented during the COVID-19 pandemic.

**Methods:**

The Twitter profile @HospitalsTalkTo and hashtag #HospitalsTalkToLovedOnes were created to interact with the Twitter public between 7 June 2021 and 7 February 2022. Conversations surrounding #HospitalsTalkToLovedOnes were extracted and subjected to natural language processing analysis using term frequency and Markov chain analysis. Qualitative thematic analysis was performed on the 10% most interacted tweets and of tweets mentioning “COVID” from a personal experience-based subset.

**Results:**

We collected 4412 unique tweets made or interacted by 7040 Twitter users from 142 different countries. The most frequent words were patient, hospital, care, family, loved and communication. Thematic analysis revealed the importance of communication between patients, patients' loved ones and hospitals; showed that patients and their loved ones need support during a patient's hospital journey; and that pediatric care should be the gold standard for adult care. Visitation restrictions due to COVID-19 are just one barrier to communication, others are a lack of phone signal, no space or time for asking questions, and a complex medical system. We formulate 3 recommendations to improve the inclusion of loved ones into the patient's hospital stay.

**Conclusions:**

“Loved ones are not ‘visitors' in a patient's life”. Irrespective of COVID-19, patient's loved ones need to be included during the patient's hospital journey. Transparent communication and patient empowerment increase patient safety and improve the hospital experience for both the patients and their loved ones. Our findings underline the need for the concept of family-centered care to finally be implemented in adult nursing clinical practice.

## 1. Background

Hospitals are institutions whose primary task is treating patients, with specialized care given by expert healthcare teams. While the patient is the focus of the healthcare team, their loved ones (family/relatives/friends) also require attention. The inclusion of loved ones in a manner that allows collaboration between the patient, their loved ones, and the healthcare team is recognized in both the family-centered care (1) and shared decision making (2) models of healthcare provision. While originating in pediatrics (3), the value of family-centered care has also gained recognition in the adult care setting, with the Society of Critical Care Medicine releasing guidelines for family-centered care in the ICU (4) and first attempts being made toward developing a universal model of family-centered care (5). Furthermore, direct support from physicians and nurses for patients' loved ones is very important, with support strategies having been shown to reduce prolonged grief symptoms for relatives of patients dying in the intensive care unit (6).

In 2020, at the start of the global COVID-19 pandemic, hospitals implemented strict visitation restrictions intended to minimize hospital traffic and the spread of the virus. Their implementation created a situation which nullified the concept of family-centered care (7). In response, US and Europe created guidelines and toolboxes to uphold the standards of family-centered care (8) and family involvement (9) respectively. For infants and their parents and caregivers, there was also strong advocacy for a zero-separation policy in response to COVID-19 visitation restrictions (10).

The social distancing measures associated with the COVID-19 pandemic resulted in the significant shift to digital communications. Many conversations were transferred to a variety of social media channels, such as Twitter. Twitter allows users to create their own content, disseminate content from other Twitter users or other online material, and participate in discussions related to specific tweets or hashtags (#). All content can be publicly shared and read, while the use of hashtags makes the content searchable and discoverable and allows communities to be built around topics of interest, e.g., disease-specific hashtags about cancer care (11). While the first papers on the use of Twitter for health-related research were published in 2009, the publication count has increased rapidly since 2015 (12). Twitter can be used as a tool for promoting healthcare advocacy (13), gathering opinions on health topics through surveys (14), analyzing behavioral patterns within the society (15), and disseminating healthcare research through the use of hashtags (16). It is also a very useful tool for public health research using methods such as content or network analysis (17).

Including lived experience and public opinion into research improves quality and impact of the research (18). Family-centered care is predominantly described from the healthcare and clinical significance perspective (1, 4, 5, 8, 19) while directly from the personal experience point-of-view, we found only one study from 2014 describing the inclusion of a daughter into her mother's hospital stay, however in a not so positive way (20). The main goal of this study was to explore the public's experiences of and opinions on communication between hospital-based healthcare providers and patients' loved ones, as related but not limited to the rigorous mitigation measures implemented during the COVID-19 pandemic.

## 2. Methods

### 2.1. Campaign development and outreach

This study was designed to explore the publics opinions regarding all aspects of priority to loved ones in a patient's hospital journey by leveraging Twitter as an easy tool for widespread outreach for crowdsourcing studies. We therefore conducted a campaign on Twitter to share relevant content regarding our research question and to actively engage in accruing discussions to explore the Twitter publics opinion more in-depth in a crowdsourcing style. We created the Twitter profile @HospitalsTalkTo to use as a professional front for the campaign and to share content using #HospitalsTalkToLovedOnes on the topic of involving loved ones in a patient's hospital journey. The campaign was conducted from 7 June 2021 to 7 February 2022 (end of the 3rd wave through to the middle of the 4th wave of COVID-19, northern hemisphere). Shared content included own material, relevant tweets of other Twitter users regarding our research question, relevant news, articles and other informational content and scientific papers (Supplementary Table S1). The Twitter profile was managed by MH while the other authors of this paper were asked to promote the visibility of #HospitalsTalkToLovedOnes. Occasionally, other Twitter users whose profiles indicated a connection to the healthcare setting were tagged in tweets as a means of gathering their opinion and increasing interaction and visibility within the Twitter community.

### 2.2. Data extraction

Using the Twitter API, tweets containing #HospitalsTalkToLovedOnes as well as the entire resulting conversation (i.e., all replies and quotes, as well as the replies and quotes to those tweets) created during the study period, were fetched. The following parameters were collected for each tweet: hyperlink, date of creation, rendered content, unique ID of the tweet, conversation ID (the unique ID of the first tweet in a thread), number of replies, number of likes, number of quotes, hashtags used, links to other websites, all media data and username of the author of the tweet.

Furthermore, the following information about the user who created the tweet and the users who responded to the tweet (replied, liked, retweeted or quote-tweeted) was collected for each tweet: username, unique user ID, joining date of the user, location as provided by the user, number of followers and number of accounts the user follows.

In addition to the set of all tweets, a subset of tweets was compiled which contained only tweets that were created by other Twitter users (excluding authors of this paper to ensure an unbiased view) in response to a tweet by @HospitalsTalkTo carrying #HopsitalsTalkToLovedOnes. We used the parameter “links to other websites” to determine whether a tweet included a Twitter link or a link to another website. Differences between both datasets were tested using the Chi-squared test at an alpha of 0.05 using R software (21).

### 2.3. Data cleaning and analysis

Tweet processing, natural language analysis and further downstream analysis were conducted using R Software. Initially, retweets and tweets which contained only emojis, hashtags, hyperlinks and user references were removed. Then emojis, hashtags, hyperlinks, user references as well as names, academic titles, numbers, punctuation and common stop words [retrieved from the R package tidytext (22)] were removed from within each tweet. Words were manually harmonized by the authors to their infinitive (e.g., agrees/agree) or singular form (e.g., doctors/doctor) before the absolute and relative frequencies of single words and bigrams were calculated for both datasets, results were represented as word clouds.

Furthermore, from the dataset containing all tweets word bigrams were extracted and subjected to network analysis where each node represents a single word being part of at least one of the extracted bigrams and each directed edge the connection of the first and second word of these bigrams with opacity indicating absolute frequency of bigram occurrence. This results in a Markov chain display where the point of each arrow of each word depends on its previously occurring word. We conducted this Markov chain network representation of the most commonly occurring bigrams (more than three times) using the R package tidytext (22) as described in (23).

A smaller subset of tweets containing conversations in response to tweets shared by @HospitalsTalkTo only, was subjected to qualitative content analysis following Braun and Clarke (20). Thematic analysis were undertaken for tweets containing the word “COVID” and for the top 10% of the most interacted tweets (=sum of replies, likes, retweets and quote tweets). Two researchers (MH and AT) independently read the tweets and identified categories for the parts that were relevant for involving loved ones in a patient's hospital journey. This restrictive evaluation was carried out due to the large number of tweets. Main, overarching topics were identified to which the tweets were then allocated. A tweet could be allocated to more than one theme. Finally, the selected categories were compared again with the tweets to ensure that no important topic was overlooked.

User data was summarized using median and IQR (Interquartile range). Their locations were harmonized to the country level by the authors. Where more than one location was listed, only the first one was considered.

### 2.4. Ethical approval and informed consent

As Twitter is a public platform where the users agree to share their activity publicly, no informed consent or ethical approval was needed. We do not provide any account names or other personal information which might allow the possibility of individual identification.

## 3. Results

### 3.1. Tweet volume and interacting users

During the campaign, a total of 4,412 unique tweets were posted that used #HospitalsTalkToLovedOnes or were created as a response to a tweet carrying the hashtag. A total of 7,040 Twitter users created or interacted with the tweets and the Twitter profile @HospitalsTalkTo gained 1,045 followers during that time.

**Figure 1:**
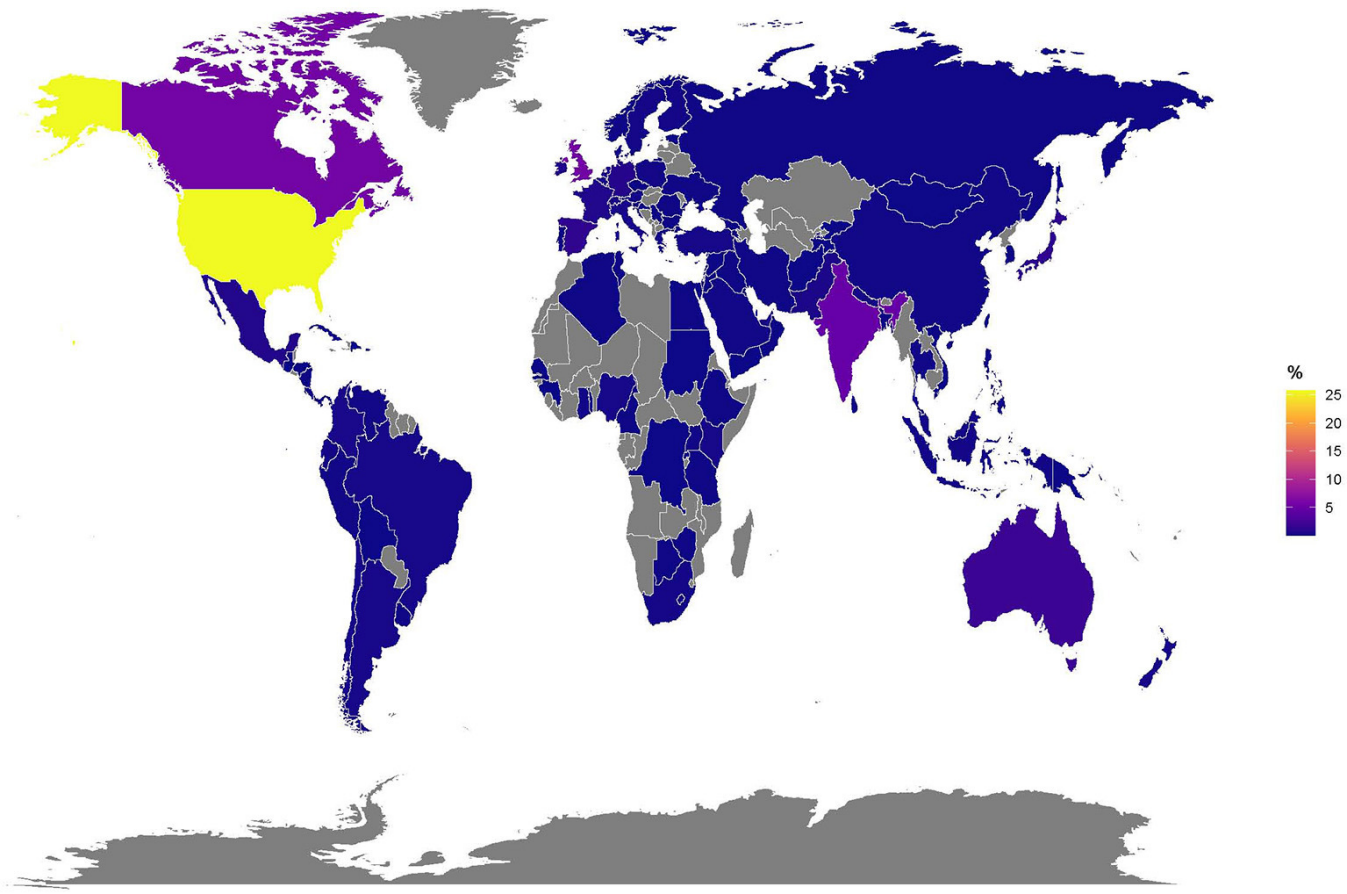
Locations of users who interacted with @HospitalsTalkTo or #HospitalsTalkToLovedOnes on a world map (*N* = 7040).

The interacting users had a median of 704 (IQR= 207.75–2268) followers and were following a median of 974 (IQR= 371–2495) other users. They have posted a median of 6,492 (IQR= 1,168.0–30,295.5) tweets and spent a median of 2,450 (IQR= 1,018.25–3,706.0) days on Twitter before the first tweet containing #HospitalsTalkToLovedOnes was posted. Of 7,040 users, 4,361 stated their location. Altogether, users from 142 different countries interacted with the hashtag or related tweets (Figure 1). Most users (≥1% of users) originated from the US, followed by the UK, Canada, India, Australia, Japan and Spain.

### 3.2. Source content in tweets

In the total tweet set, 41% (1,799/4,412) of tweets contained only text (own content), 37% (1,620/4,412) of tweets contained a link to a source originating from Twitter (another tweet) and 23% (993/4,412) contained an external source from the internet (scientific paper, news article, etc.). In the @HospitalsTalkTo dataset, 73% (587/806) of tweets contained only text, 25% (205/806) contained links to sources on Twitter and only 2% (14/806) contained links to external sources. All named frequencies were significantly different between the two data sets (*p* < 0.01).

### 3.3. Word frequency analysis

**Figure 2:**
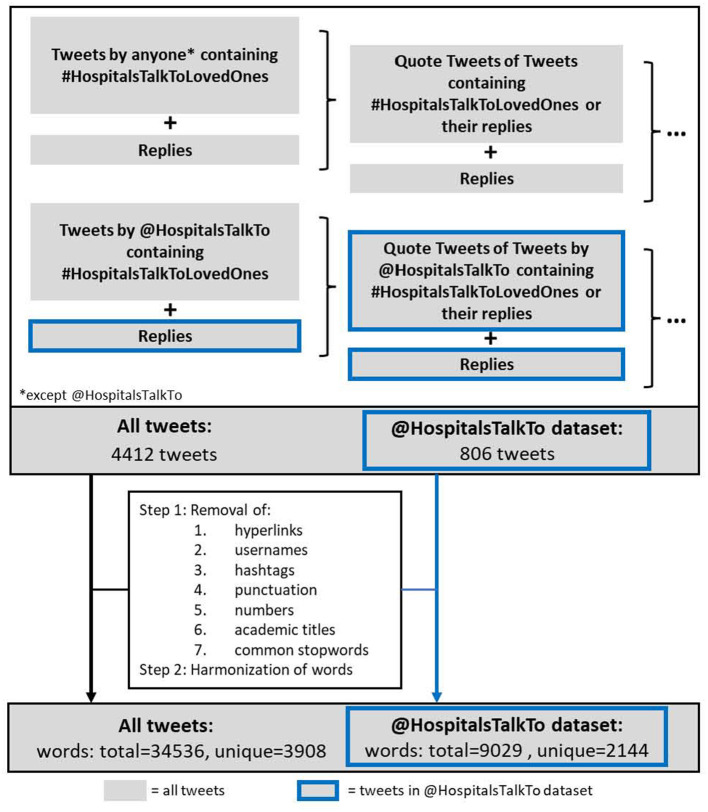
Tweet analysis flowchart. All tweets (=4412, gray) and tweets within the @HospitalsTalkTo dataset (=806, blue) were subjected to clean-up processing, resulting in 34536 words overall (3908 unique) for all tweets, and 9029 (2144 unique) within the @HospitalsTalkTo dataset.

**Figure 3:**
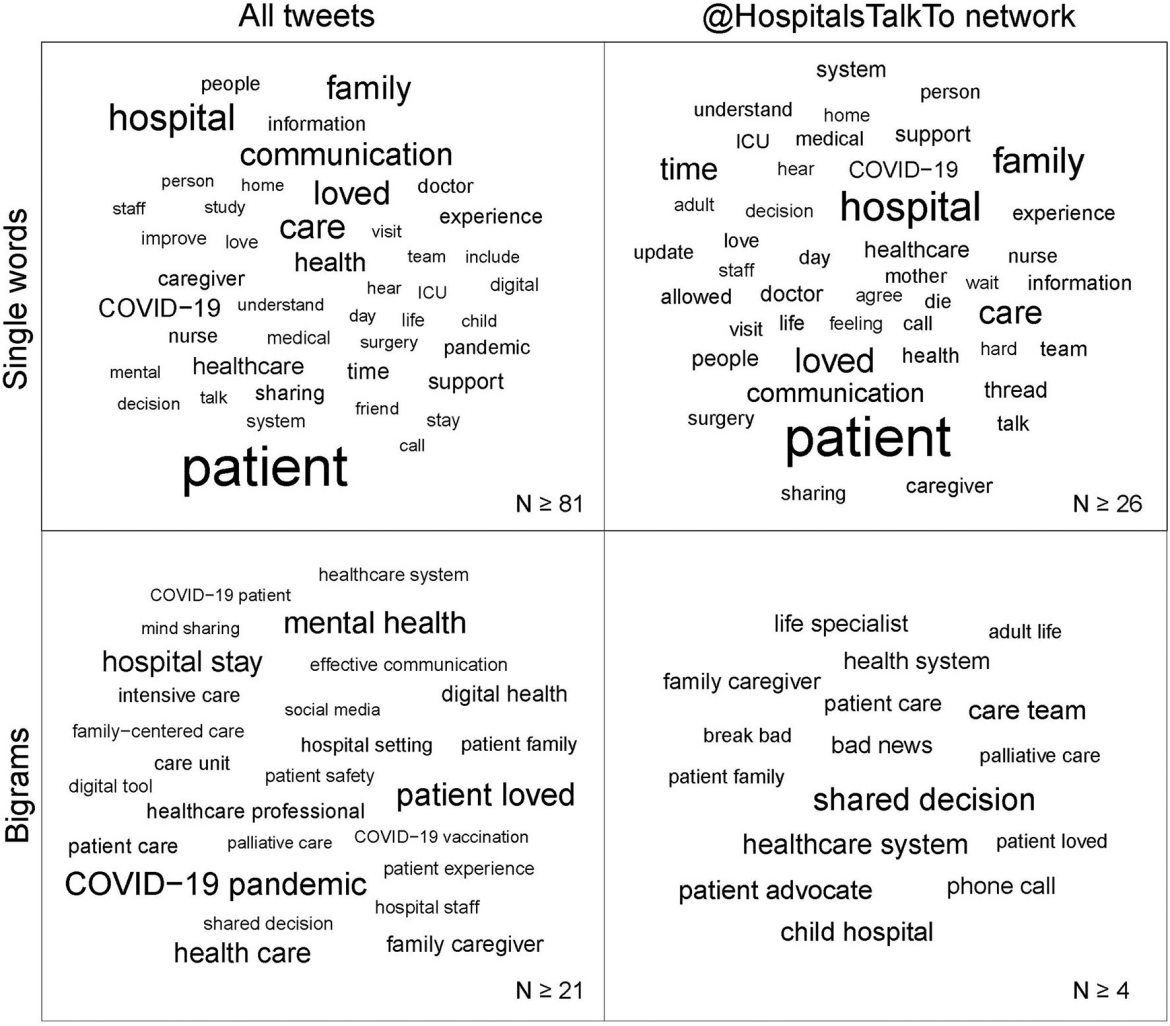
Word clouds for items among all tweets and within the @HospitalsTalkTo dataset. Size of the words is proportional to word frequency. The thresholds for inclusion are written in the bottom right corner of each square.

To gain more detailed insight into the content shared about #HospitalsTalkToLovedOnes, the tweets were analyzed by a language processing algorithm (see Methods: Data cleaning and analysis). The whole dataset yielded 3,908 unique words from a pool of 34,536 words; the @HospitalsTalkTo dataset yielded 2,144 unique words from a pool of 9,029 words (Figure 2). Within both datasets, the most frequently used words were “patient”, “hospital”, “care”, “family”, “loved”, and “communication”, which present a good summary of #HospitalsTalkToLovedOnes (Figure 3). The @HospitalsTalkTo dataset also included the word “time” among those most frequently used, and was also very abundant in all tweets. In both datasets, other frequently occurring words were “experience”, “information”, “understand”, “visit”, “COVID-19” and words related to the reason for communication (“hear”, “support”), type of communication (“call”, “talk”), healthcare team (“doctor”, “nurse”), affected persons (“caregiver”, “child”, “mother”, “friend”), type of hospital stay (“surgery”, “ICU”), health status (“life”, “die”) and emotion (“love”, “feeling”).

The most common bigram among all tweets was “COVID-19 pandemic”, among the very common were also “COVID-19 patient” and “COVID-19 vaccination”. The most common bigrams within the @HospitalsTalkTo dataset were “shared decision”. In both datasets, there were bigrams related to the hospital system (“patient care”, “hospital staff”). Among all tweets, healthcare concepts (“family-centered care”, “digital health”, “patient safety”, “patient experience”, and “shared decision”) were frequently mentioned. The @HospitalsTalkTo dataset contained bigrams related to the type (“phone call”) and content (“break bad”, “bad news”) of communication and personas to communicate (“care team”, “life specialist”, “patient advocate”) displaying a more personal experience-based Twitter communication.

### 3.4. Markov chain analysis

**Figure 4:**
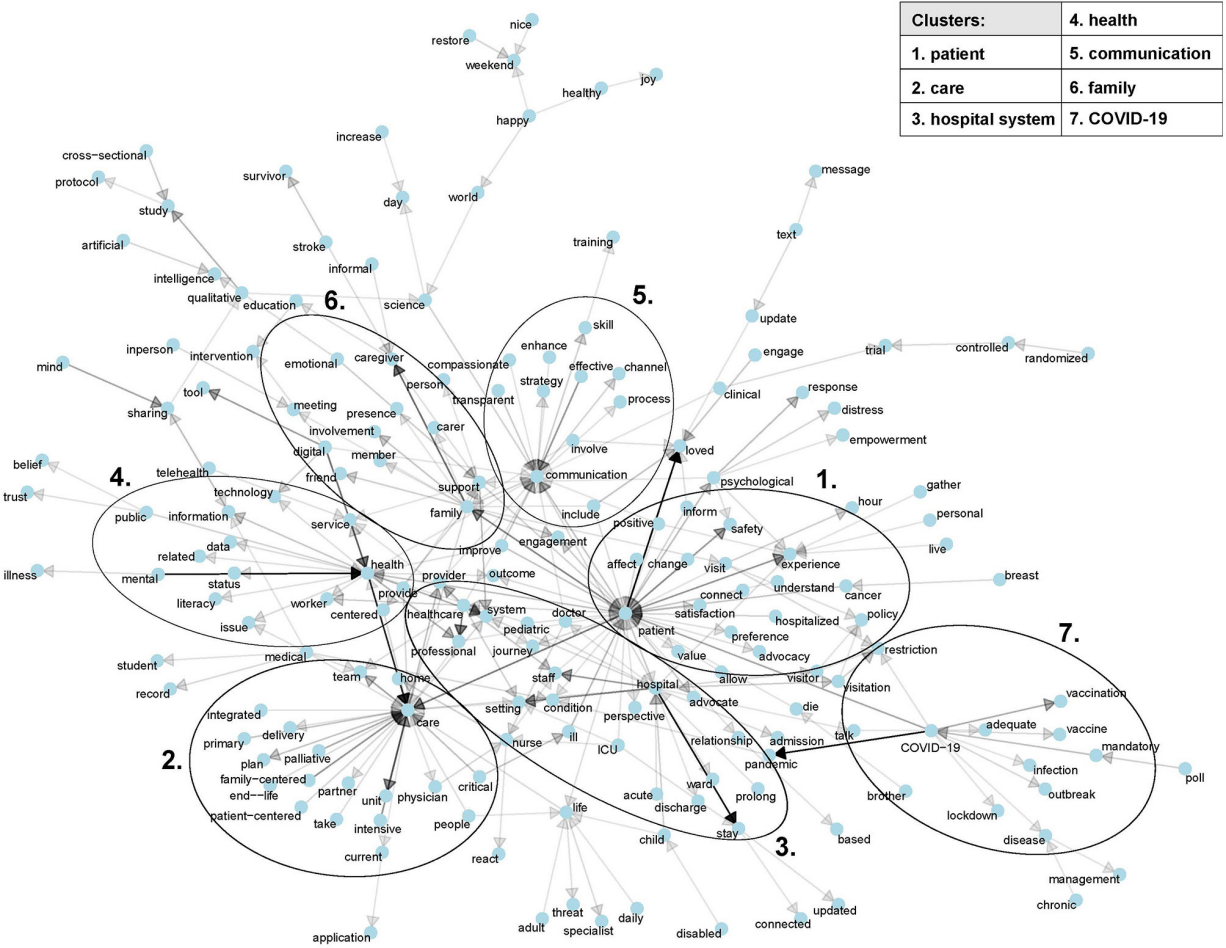
Markov chain analysis of bigrams occurring more than three times among all tweets. Networks consisting of six or fewer items were omitted. The seven clusters of the major network hubs were defined by the authors based on thematic background and connectivity.

Markov chain analysis with network representation of connections between frequently occurring words within the whole dataset was done. In accordance with the largest word hubs, seven clusters were identified with respect to the thematic background and connectivity of these hubs. The central cluster is the hospital system with its most important players: nurses and doctors. It is surrounded by clusters relating to patients, loved ones, care, communication, health, and COVID-19 (Figure 4).

### 3.5. Qualitative analysis of the top tweets within the @HospitalsTalkTo dataset

Qualitative analysis of the top 10% tweets (= 81 tweets of 806) that gained between 22 and 928 interactions within the @HospitalsTalkTo dataset revealed seven prominent themes (Table 1). Seven tweets bore no relevance to healthcare (e.g., “*I'm such a cliché*”).

**1. Communication between hospitals and loved ones is important (32/81)**.

**Table 1:** Tweets representative of the seven main topics mentioned in the top 10% of tweets within the @HospitalsTalkTo dataset.

**Communication between hospitals and patients' loved ones is important (32/81)**
• “If you're a doctor reading this, be the bridge that connects your patients to their families […]” **Barriers of communication** • “It was probably an innocent oversight, but this turned into a traumatizing problem for me during what was already the most traumatic event of my life. Please, never make anyone wait alone with no signal in a situation like this.” • “As a nurse I'm happy to talk to the family, but I'm often so busy that I have to schedule it. […]” **How to improve communication** • “[…] It also helps if there's a single point person & not 4-6 family all calling at different times asking for the same info.” **Digital communication tools** • “When our son had surgeries (children's hospital) there was an app that provided text updates as well as a person that would go between ORs and waiting room to update. It was wonderful! […].”
**Needing/finding support as a loved one (24/81)**
• “Loved ones are not ‘visitors' in a patient's life.” • “Don't be afraid to call and ask! It may take all night, but you have the right to know what's going on” • “1. Don't neglect yourself; 2. Fortify yourself emotionally; 3. Rely on others.” • “[…] It's so NOT simple to die at home: unpaid, untrained, anxiety-ridden family caregivers (who may also be the Proxy, facing push-back) get scared, overwhelmed and unable to cope.” • “No one calls to give updates. You pace and worry.” • “[…] Sitting in a waiting room most always excruciating for loved one(s). […]”
**Loved ones should have a place at the bedside (20/81)**
• “It's heartbreaking to sit at home because your loved one is taken to hospital by ambulance and you not allowed in. […] We lost our precious last hours being apart. No final words of comfort.” • “I was hospitalized for open heart surgery when the pandemic first hit in 2020. Imagine, I had to take an Uber to the hospital at 4:30 a.m., by myself, as no family could enter the hospital. When I woke up in recovery, no one was there waiting to see me. […].” • “I sometimes wonder if the PTSD from all my ICU stays would be significantly less severe if my loved ones were always allowed to be there.” • “When I was in the ICU, the hospital allowed […] to come, sit with me and talk to me. He was in full PPE and just his presence made a world of difference to me emotionally & am sure that helped with my recovery too. Please allow loved ones.” • “Having a #Caregiver or friend at the bedside to advocate or soothe is essential to patients' health. […]”
**The importance of human interaction (19/81)**
• “[…] As humans our relationships sustain us, help us grow, heal, feel safe. […]” • “[…] A personal touch is very important apart from the treatment given by doctor!” • “Communication during care is important also because it helps the grieving process. If our doubts/fears/questions are addressed we have more peace (trust) overall which in the long run is good for everyone involved.” • “[…] Cut off from loved ones, my mother was allowed to see me for just 15 mins. Seeing her though made the world of difference.”
**Healthcare system should support patients more (18/81)**
• “I was shocked […] by how isolated and powerless I felt as a patient. And this was despite the fact that I was a physician at the hospital where I was admitted! We need to do more to support and empower our patients.” • “[…] When you are dependent on care and you don't get the info and support you need, when the healthcare system doesn't listen to your concerns, it's a betrayal of trust. […]” • “[…] we need adult life specialists who help us not traumatize adults with procedures and help people cope with hospitalization!” • “I think the reality is that when you or a loved one are sick the medical system is a complex, confusing, and scary place and we don't do enough to acknowledge and help with that”
**Communication with patients is important (11/81)**
• “When patients apologize for “bothering” me, I feel awful; experience taught them I'll be annoyed or think their concerns are foolish. No. They trusted our care team to take good care of them; we need to see the job through.” • “Clear picture painted for them about what it will be like going home, how will the illness behave, what will be the anticipated hurdles & milestones. How to prepare for the twists & turns of the illness. Honest, realistic, informed picture.” • “[…] Knock, introduce yourself, describe what you are there to do, sit/slow down. Applies to all settings!.” • “Talk less….listen more. This is the space where the real stuff happens.”
**Adult care should strive to be more like pediatric care (8/81)**
• “[…] As a pediatrician I'm often struck by how I wish my adult care was more like pediatrics” • “[…] there are many ways that adults ARE just big kids! We in the grown-up world need to be better at considering the whole person, the social context, and emotional suffering.” • “I once had to get imaging at a children's hospital […]. A child life specialist came and explained to me what was going to happen and what the contrast injection would feel like. It was amazing!”

Quotation marks represent (excerpts) of original tweets. Some tweets were corrected for grammar.

Problems in obtaining information about a patient in the hospital were frequently mentioned. Barriers in communication were: “*no signal in hospital rooms”* and “*hospital staff not having much time for giving updates”*. A suggestion for improving communication was “*there only being one designated loved one to communicate information to”*. Digital communication tools were mentioned as being useful, e.g., an app that provides text updates on the status of surgery.

**2. Needing/finding support as a loved one (24/81)**.

A patient's hospital stay was reported as being very emotional and stressful for loved ones (“*traumatic experience”, “pace and worry”, “excruciating”*). There was a consensus that loved ones should be involved in a patient's hospital stay as “*Loved ones are not “visitors” in a patient's life*”. They are emotionally invested themselves and need support in their role.

**3. Loved ones should have a place at the bedside (20/81)**.

The hardship of not being able to be at a patient's bedside when they are dying was recounted. Patients reported feeling frightened when waking up after surgery alone or wondering whether the PTSD from an ICU stay would be less severe with loved ones more present. There was an appreciation for hospital systems that allowed visitation.

**4. The importance of human interaction (19/81)**.

The value of personal interaction was indicated as it can create the feeling of safety and value and can make a world of difference.

**5. Healthcare system should support patients more (18/81)**.

The focus was also on patients and their need to experience more support *(“we need adult life specialists”*) and empowering hospital healthcare professionals so they can trust and rely on the medical system and know how to navigate it in all its complexity.

**6. Improving communication with patients (11/81)**.

Users stated that patients shouldn‘t feel bad when asking questions and should gain a realistic expectation of the disease management and outcomes. Clear suggestions were offered regarding necessary communication with patients: “*Knock, introduce yourself, describe what you are there to do, sit/slow down.”, “Talk less….listen more”*.

**7. Adult care should strive to be more like pediatric care (8/81)**.

Pediatric care was used as an example of how adult care should be, with Twitter users providing only positive examples of how pediatric care includes families (loved ones) and is sensitive to the patient's needs.

### 3.6. COVID-19 effects on communication

Qualitative analysis of tweets explicitly mentioning “COVID” within the @HospitalsTalkTo dataset yielded 41 (of 806) tweets, which were allocated to three main themes (Table 2). Five tweets were not related to communication.

**1. Visitation restrictions: implications for loved ones (24/41)**.

**Table 2:** Tweets representative of the three main topics which mention COVID-19 within the @HospitalsTalkTo dataset.

**Visitation restrictions: implications for loved ones (24/41)**
• “One of my patients lost her fiancé to COVID. He had been admitted to the hospital. She could only visit once a day for 15 mins. When he was admitted to ICU she could no longer visit him. He had to die alone, and she never got to tell him goodbye. She needed to see him.” • “[…] Hospital had rules for visitation of COVID patients. Nobody was allowed to. We even tried through interns there to check […]” • “[…] luckily allowed to visit as a special exception was granted due to my being a physician. No other visitors were allowed due to COVID19 restrictions. […]” • “I recently had surgery, COVID rules meant he wasn't allowed to set foot inside, either to drop off or collect. This broke the handover process on “how to find out how your loved one is going” […].”
**Visitation restrictions: implications for patients (13/41)**
• “A 80+ year old patient of mine said he'd rather die than go back into hospital after being in last year (non-COVID) during time of no visitors. He felt like he was going to go crazy not being able to see people. […]” • “Sometimes they need to see a friendly face and someone to care. Hospitals are short staffed, and doctors have little time to spend with patients. […]” • “[…] If a person has depression or anxiety being alone while being hospitalized makes it worse. […]” • “My near deaf husband in SICU during COVID couldn't communicate clearly with staff who didn't know how to work with him. Had I been allowed to be there the staff would have benefited from my help. Instead he was traumatized & terrorized by one nurse who lost it”
**Visitation restrictions: implications on communication (13/41)**
**Personal communication by healthcare professionals** • “During the peaks of COVID-19 our ITU had a senior nurse each day dedicated to phoning relatives and giving them updates.” • “I always get permission before talking to family members; a lot of times COVID patients don't want us talking. […]” • “The surgeon forgot to call my mom to let her know the surgery went OK, and my aunt was too sedated to be able to call. We did not know if she was alive for ~24 h.”
**Communication technology**
• “[…] I relied on video call to connect to my dad regularly and make him emotionally fit.” • “[…] They had a texting service with updates and it was such an anxiety reducer for me especially in the age of COVID-19. […]”

Quotation marks represent (excerpts) of original tweets. Some tweets were corrected for grammar.

Twitter users described the hardship of hospital visitation restrictions. They reported not being able to see patients at all, trying to gather information through interns, or only being granted access because they themselves were doctors.

**2. Visitation restrictions: implications for patients (13/41)**.

A Twitter user reported that her patient would “*rather die than go back into hospital”*. Another reported problems occurring due to her not being at the bedside to advocate and translate the patient's needs to the healthcare team, which led to severe patient safety issues. Not being able to have visitors is increasingly hard on patients with depression and anxiety.

**3. Visitation restrictions: implications on communication (13/41)**.

Some reported special measures adopted to inform patients' loved ones, such as a nurse designated to only communicate with loved ones, and nurses getting instructions from patients about what to communicate. However, there were also negative examples of physicians forgetting to update the loved ones, resulting in “*We did not know if she was alive for 24h”*. Communication was by text, video and phone call.

## 4. Discussion

This study underlines the importance of communication between hospital-based healthcare professionals and a patient's loved ones in the context of, but not limited to, COVID-19-related hospital visitation restrictions. The main findings are: firstly, the need for communication between hospital-based healthcare professionals and a patient's loved ones is global. Secondly, transparent communication and human interaction are an important part of a healthcare system and can be supported by digital communications. Thirdly, pediatric care should be the gold standard for adult care as a model of incorporating loved ones into family-centered care. Finally, hospital visitation restrictions are harmful both to patients and their loved ones, with patients feeling lonely, not having their loved ones to advocate and explain their needs to the healthcare team, or even refusing to go to hospital to seek treatment.

The experience of loved ones, as well as patients, is strongly influenced by “mepathy”, i.e., it is only after the personal experience of a hospital stay that the problems and potential areas of improvement are acknowledged. Moreover, the contribution of loved ones to patient care usually goes unrecognized (24). Testimonials based on personal experience of the importance of including loved ones have been published by impactful medical journals of different specialities:

“*Through my family tragedy, it became clear to me that the environment in our ICUs often serves the convenience of the staff who work in the ICU, rather than the critically ill patients and their loved ones who are, as a family unit, the objects of our care.”* Dr. Levy, Critical Care Medicine, 2007 (25)

“*Those of us who have survived trauma need our healthcare providers to meet us in our Quiet Place. We need them to find their way into that dark chamber, light a candle, and fill it with the words that build a bridge for us to walk out.”* Ms. Flanary, MA (wife to internet comedian Dr. Glaucomflecken), Journal of Cardiac Failure, 2021 (26)

Currently, efforts are being focused on meeting the needs of the loved ones of patients on intensive care units. Although missed opportunities are common (27), systematic support strategies for loved ones are being developed to change this situation (6). In the US, the needs of loved ones are also addressed during the time surrounding surgery (28–30). However, as our data and the literature (20) shows, most loved ones' needs are not being met and are often overlooked, especially outside the intensive care setting.

### 4.1. COVID-19 effects on inclusion of loved ones into hospital stays of patients

Strict visitation restrictions during COVID-19 impacted the hospital care and were discussed on Twitter. Our data shows that alternatives to personal communication at bedside with the aim to include loved ones into patients' hospital stays were varying. There were reports of designated nurses in charge of communicating with loved ones, of failures in communicating that a surgery went well and subsequently loved ones not knowing for 24 h if the patient was alive, and of a patient saying they would rather die than go back to the hospital. Overall, visitation restrictions were traumatic and had negative influences on all involved: patients, loved ones and hospital staff (9, 31–34) and limited to nullified the possibility of providing family-centered care (7, 9). Instead of complete visitation restrictions, visitations should be treated as a limited yet highly important resource (7) and independent committees should be allocated to manage them (35, 36).

Family-centered care needs to adapt to include strategies regarding the inclusion of loved ones that are not physically present at bedside, either due to pandemic conditions (31, 36), seasonal influenza (37) or lack of means or opportunities on the side of loved ones. This was the first pandemic where digital and telehealth tools were used to support phone-call based communication. Virtual visiting was shown to reduce loved ones' anxiety, benefit patient recovery and staff morale (32, 38). It seems only reasonable for hospitals to invest in and routinely adopt digital and telehealth tools to uphold and offer robust and inclusive family-centered care irrespective of the circumstances.

### 4.2. Recommendations for better including patient's loved ones in the hospital stay

Based on the experiences and wishes gathered through our #HospitalsTalkToLovedOnes campaign, we have formulated three recommendations to establish better communication between hospitals and patients' loved ones.

**1. Establishing a reliable communication channel and allowing loved ones at the bedside**.

The Twitter community provided suggestions for better including patients' loved ones: by guaranteeing a stable phone connection in areas where patients or loved ones are waiting or staying; having only one designated loved one to manage all communication; and sending text updates. There was strong advocacy for allowing loved ones at the bedside, their absence being associated with anxiety, fear, PTSD, and mourning the missed opportunity to say goodbye. Studies from the intensive care unit from the perspective of patients, loved ones and healthcare professionals (39, 40) support an open visitation policy. We are aware of the significant pressure hospitals are under, both during COVID-19 and on an everyday basis, however, in hindsight and going forward, hospitals should prioritize and allocate staff to managing communication with loved ones. One example given by Twitter users was having a nurse dedicated to phoning relatives and giving them updates. Special bespoke teams have been positively accepted by loved ones during COVID-19 (41). Furthermore, creating room for communication with loved ones also positively affects the healthcare team (42).

**2. Embracing digital communication tools**.

Irrespective of COVID-19 visitation restrictions, strategies are needed to involve loved ones who cannot be physically present in the hospital (due to work, distance, personal reasons, etc.). Even before COVID-19, in the US digital communication between hospitals and a patient's loved ones took place around the time of surgery through the use of perioperative messengers (30, 43). Further development of digital communication tools (44, 45), virtual visiting options (32, 38) and patient portals (46–48) has huge potential to help alleviate non-communication or support current forms of communication both with patients and loved ones. However, in purely online communication, attention must be paid to the quality of communication, as the quality of diagnosis information exchange affects patient initiative and the quality of physician treatment recommendations (49).

**3. Applying the principles of pediatric care to an adult care setting**.

People want adult healthcare to be more like pediatric care. While pediatric care relies on family-centered care (19) and shared decision making (50), adult care requires a high degree of patient autonomy and independent skills, and provides few interdisciplinary resources and support (51). The contrast is clearly demonstrated during the transition from pediatric to adult care (52, 53). To a large degree, our results coincide with the guidelines created to apply family-centered care at the neonatal, pediatric and adult ICU (4). Our study supports the need for family-centered care in the adult setting, not just from the perspective of loved ones' involvement (5), but also to achieve a more holistic approach which considers all the patient's needs—physical, social, and emotional. As navigating any healthcare system is complex and confusing, Twitter users raised the idea of implementing adult life specialists: these act as a support person, explain the proceedings to the patient and their loved ones, and advocate for the patient in the hospital. This idea is based on child life specialists—professionals who work with children, helping them understand and cope with illness or hospitalization and striving to alleviate their stress and anxiety (54, 55). Support strategies as described by Kentish-Barnes et al. (6) are a step in that direction, although such strategies also need to be developed outside the end-of-life, intensive care setting.

### 4.3. Strengths and limitations

One of the major strengths of our study is the integrative knowledge and experience transfer between individuals from all over the world. Online communities on Twitter can serve as a source of health information transfer and practice exchange (56). Social media campaigns, including on Twitter, are also likely to improve care for patients (57). However, there are some limitations associated with Twitter studies. First, a self-selection bias is unavoidable as only Twitter users can interact and contribute to the conversation. Secondly, there is no transparency as to who see the tweets and to whom the Twitter algorithm promotes the tweets in the Twitter feed. Twitter hashtag communities provide more transparency and clustering of topics, however, one needs to know the hashtag to be able to search for it. Thirdly, our study is limited in its power to express the content of tweets using single words and bigrams with respect to the holistic experiences and opinions shared. This we have counteracted by applying a qualitative thematic analysis of tweets and providing direct quotes from those tweets. Lastly, there are no established success metrics for social media studies, and a scoping review from 2021 identified only a few studies on the public health community's use of social media for policy advocacy over the last decade (58). Our study combines views of patients, loved ones and hospital based healthcare professionals, achieving international interaction. The study strongly supports active communication with and integration of loved ones into the patients' hospital stays.

### 4.4. Conclusion

“*Loved ones are not ‘visitors' in a patient's life”* and hospitals must include them in the patient's hospital journey. Our data shows the public's experiences regarding not only but also COVID-visitation restriction related loved ones' involvement in hospital stays of patients and wish for more inclusion, transparency, communication, and importance of being at bedside, which to a high degree overlaps with the objectives of family-centered care. We conclude that while the theoretical basis is already in place, family-centered care is lacking in application. Finishing with a statement by a Twitter user, “*If you're a doctor* [or any kind of hospital-based healthcare professional or decision maker] *reading this, be the bridge that connects your patients to their families”*.

## Data availability statement

The raw data supporting the conclusions of this article will be made available by the authors, without undue reservation.

## Ethics statement

Twitter is a public platform where the users agree to share their activity publicly, therefore no informed consent or ethical approval was needed. We do not provide any account names or other personal information which might allow the possibility of individual identification. Ethical review and approval was not required for the study on human participants in accordance with the local legislation and institutional requirements. Written informed consent from the participants' legal guardian/next of kin was not required to participate in this study in accordance with the national legislation and the institutional requirements. Written informed consent was not obtained from the individual(s), nor the minor(s)' legal guardian/next of kin, for the publication of any potentially identifiable images or data included in this article.

## Author contributions

MH, AGA, and ES conceptualized and designed the study and were responsible for the decision to submit the manuscript. MH was leading the Twitter campaign and MH drafted the manuscript. FAN, MC, CSS, HPD, RD, RKS, EDP, CT, and AGA supported the Twitter campaign. LK extracted the data from Twitter. MH and LK verified the underlying data. FE and MH analyzed the data. FE, MH, AT, and ES interpreted the data. All authors had full access to all the data in the study, had final responsibility for the decision to submit for publication, participated in the critical review of the manuscript, read, and approved the final manuscript.
