# Supplementary material for: “Loved ones are not ‘visitors' in a patient's life”—The importance of including loved ones in the patient's hospital stay: An international Twitter study of #HospitalsTalkToLovedOnes in times of COVID-19
Source: Front Public Health. 2023 Jan 26;11:1100280. doi: 10.3389/fpubh.2023.1100280 (PMC9909431; doi:10.3389/fpubh.2023.1100280)
Supplement: Supplementary file 1 [file Table_1.pdf]

Supplementary Material

Table S1: Examples of content shared by @HospitalsTalkTo. Usernames have been removed to assure complete anonymity of tweets.

Type of content shared by @HospitalsTalkTo	Examples
Own content	“Within the #HospitalsTalkToLovedOnes initiative, I spoke to @[redacted]<br>about her experience of #Canada’s healthcare system as a #caregiver to her husband<br><br>Whole interview: https://rb.gy/euor4f<br><br>Lots of great insights, thank you for sharing! 🙏 #EssentialCarePartner #MedTwitter”<br><br>https://twitter.com/HospitalsTalkTo/status/1460981137908637706
Relevant tweets by other Twitter users	“It is so important that loved ones are included in a patients healthcare journey.<br>We need #HospitalsTalkToLovedOnes❣<br><br>Thank you @[redacted] for sharing your story 🙏<br><br>More about the 💔 story of @DGlaucomflecken health that led to this in 🧵 below 👇 ”<br><br>https://twitter.com/HospitalsTalkTo/status/1485299327702638592
Relevant news, articles, and other informational content	“🧵 Breaking bad news over the phone: SPIKES model<br><br>👉Setting up<br>👉Perception<br>👉Invitation<br>👉Knowledge<br>👉Emotions<br>👉Strategy & Summary<br><br>Practical example by @[redacted] 👇 🤩”<br><br>https://twitter.com/HospitalsTalkTo/status/1483808552934416384
Scientific papers	“3 step strategy to support loved ones of dying patients<br>👉family conference to prepare the relatives for imminent death<br>👉ICU-room visit to provide active support<br>👉meeting after patient’s death to offer condolences and closure<br><br>💯#HospitalsTalkToLovedOnes<br>https://www.thelancet.com/journals/lancet/article/PIIS0140-6736(21)02176-0/fulltext ”<br><br>https://twitter.com/HospitalsTalkTo/status/1486077791527485447
